# MicroRNA-142-3p Negatively Regulates Canonical Wnt Signaling Pathway

**DOI:** 10.1371/journal.pone.0158432

**Published:** 2016-06-27

**Authors:** Tanyu Hu, Krung Phiwpan, Jitao Guo, Wei Zhang, Jie Guo, Zhongmei Zhang, Mangge Zou, Xuejie Zhang, Jianhua Zhang, Xuyu Zhou

**Affiliations:** 1 CAS Key Laboratory of Pathogenic Microbiology and Immunology, Institute of Microbiology, Chinese Academy of Sciences, Beijing, 100101, China; 2 College of Life Sciences, University of Chinese Academy of Sciences, Beijing, 100049, China; 3 Faculty of Allied Health Sciences, University of Phayao, 19 Moo 2 tambon Maeka Amphore Muang Phayao province, Thailand; Cleveland Clinic, UNITED STATES

## Abstract

Wnt/β-catenin signaling pathway plays essential roles in mammalian development and tissue homeostasis. MicroRNAs (miRNAs) are a class of regulators involved in modulating this pathway. In this study, we screened miRNAs regulating Wnt/β-catenin signaling by using a TopFlash based luciferase reporter. Surprisingly, we found that miR-142 inhibited Wnt/β-catenin signaling, which was inconsistent with a recent study showing that miR-142-3p targeted Adenomatous Polyposis Coli (APC) to upregulate Wnt/β-catenin signaling. Due to the discordance, we elaborated experiments by using extensive mutagenesis, which demonstrated that the stem-loop structure was important for miR-142 to efficiently suppress Wnt/β-catenin signaling. Moreover, the inhibitory effect of miR-142 relies on miR-142-3p rather than miR-142-5p. Further, we found that miR-142-3p directly modulated translation of *Ctnnb1* mRNA (encoding β-catenin) through binding to its 3’ untranslated region (3’ UTR). Finally, miR-142 was able to repress cell cycle progression by inhibiting active Wnt/β-catenin signaling. Thus, our findings highlight the inhibitory role of miR-142-3p in Wnt/β-catenin signaling, which help to understand the complex regulation of Wnt/β-catenin signaling.

## Introduction

Wnt/β-catenin signaling is an ancient, highly conserved pathway that plays pivotal roles in a wide variety of developmental and self-renewing processes. In the immune system, Wnt/β-catenin signaling is involved in the self-renewal of hematopoietic stem cells (HSCs), development and differentiation of T cells, B cells and dendritic cells (DC) [1], and whose abnormity is highly associated with tumorigenesis, such as leukemia. β-catenin, the key transcriptional coactivator of the Wnt/β-catenin pathway, is trapped and degraded in the cytoplasm by a destruction complex (encompassing GSK3β, Axin and APC). Upon activation by Wnt ligands, the subsequent phosphorylation events lead to inhibition of the APC complex-mediated degradation of β-catenin, facilitating β-catenin to accumulate and localize to the nucleus to form complex with the T-cell factor/lymphoid enhancer factor (TCF/LEF), and initiating the expression of Wnt target genes [2].

MiRNAs are small, evolutionary conserved single-stranded non-coding RNAs, which are transcribed from genome as long primary miRNA transcripts (pri-miRNA), sequentially processed from pre-miRNA to ~22 nt miRNA duplex by the Dosha/DGCR8 complex in nucleus and Dicer in cytoplasm [3], and thus yielded two mature products from each strand (termed 5p and 3p, respectively) [4–7]. Deletion of either DGCR8 or Dicer completely abrogates the biogenesis of miRNAs [8, 9]. In most cases, miRNAs inhibit gene function through direct binding to the 3’ UTR of cognate mRNAs [10].

Previous studies have shown that miRNAs also play a crucial role in T-cell development in the thymus. Dramatically decreased thymocyte number and increased thymocyte susceptibility to cell death have been found in the conditional Dicer knockout mice (Lck-Cre. Dicer^fl/fl^) [11]. Interestingly, Lck-Cre-mediated specific expression of constitutively active form of β-catenin or loss-of-function mutant of APC leads to resembling phenotypes in mice [12, 13]. The phenotype similarity between miRNA deficiency and Wnt overactivation in thymus suggested that Wnt/β-catenin signaling might be restricted by miRNAs during early T cell development. Recent studies also indicate that several miRNAs play roles in carcinogenesis through modulating canonical Wnt signaling pathway [14], for instance, miR-1, miR-25 and miR-613 inhibit the activity of the Wnt signaling [15] while miR-142-3p upregulates this signaling through targeting APC [16]. Here, we screened dozens of miRNAs highly expressed in T cells that potentially affect Wnt/β-catenin signaling pathway. Inconsistent with previous observation [16], we found that miR-142-3p downregulated Wnt/β-catenin signaling. Further validation using a number of mutants confirmed the inhibitory effect relied on the stem loop structure of pre-miR-142. Moreover, we demonstrated that miR-142-3p directly targeted β-catenin and suppressed its protein translation, thus repressed cell proliferation. Collectively, our findings provide different views of the molecular interaction between miR-142-3p and the Wnt signaling.

## Materials and Methods

### Plasmid constructs

Pre-miRNAs plus ~ 700 bp or ~ 300bp flanking sequences were amplified from mouse genomic DNA and cloned into pEF-BOS-EX vector (referred to as EF) downstream of the EF-1a promoter [17]. Plasmids del-miR-142, pre-edited-miR-142, and mutants of pre-miR-142 (M1 ~ M10) were generated using PCR directed mutagenesis of EF-miR-142 plasmid. The *Ctnnb1* 3’ UTR was inserted at the *Xba* I/*Sal* I sites of the PmirGLO vector (Promega, Madison, USA). Deletion of miR-142-3p binding sites (nt 356–361 and nt 665–671) within *Ctnnb1* 3’ UTR was generated by PCR directed mutagenesis. TopFlash DNA fragment coding eight tandem repeats of TCF response elements [18], and FopFlash DNA fragment as a negative control were synthesized and introduced to pGL3-promoter vector (referred to as pGL3) upstream of the SV40 promoter to generate pGL3-TopFlash and pGL3-FopFlash respectively. Plasmid MSCV-beta-catenin-IRES-GFP (an N-terminal ΔGSK mutation of β-catenin without its 3’ UTR) was kindly provided by Prof. Tannishtha Reya (Addgene plasmid # 14717) [19, 20]. MDH1-PGK-GFP_2.0 and MDH1-miR-142-PGK-GFP (Addgene plasmid # 11375 and # 11377, referred to as MDH1 and MDH1-142) were gifts from Prof. Chang-Zheng Chen [21]. Plasmid MO (MSCV/cmv-x-flag-IRES-EGFP/pgk-puro) was a gift from Prof. Masato Ogata, pri-miR-142 plus IRES sequence were cloned into MO to construct MO-miR142-IRES-EGFP.

### Cell culture and reagents

HEK293T cells, NIH3T3 cells, PlatE cells and Jurkat cells are conserved and subcultured by our lab. HEK293T cells, NIH3T3 cells and PlatE cells were cultured in DMEM (Invitrogen, Life Technologies, NY) supplemented with 10% FBS (Thermo Scientific, Waltham, MA), L-glutamine (Gibco, NY), penicillin, streptomycin and sodium bicarbonate at 37°C with 5% CO_2_. Jurkat cells were maintained in RPMI 1640 medium (Life technologies) with 10% FBS, penicillin, streptomycin, HEPES, β-mercaptoethanol and non-essential amino acids (Invitrogen). Lithum chloride (LiCl) was prepared as 5 M stock solutions (200 ×). Wnt3a was purchased from R&D Systems and used according to the manufacturer’s protocol. Antagomirs of miR-142-3p, miR-142-5p and control antagomir were purchased from RiboBio (RiboBio Co. Ltd, Guangzhou, China), and prepared as 20 μM stock solutions (1000 ×) using RNase-free H_2_O. Puromycin was prepared as 1 mg/ml stock solutions.

### Electransfection of Jurkat T cells

Cells were washed and resuspended at 5 × 10^7^ /ml in RPMI 1640 medium without antibiotics. Plasmid DNA (10 μg) was added to 400 μl of the cell suspensions and mixed sufficiently. After 15 minutes of incubation at room temperature (RT), cells were electroporated at 250 V, 960 μF in 4 mm electroporation cuvette using BIO-RAD Gene Pulser Xcell. Electroporated cells were rested for another 15 minutes at RT, then were transferred to prewarmed RPMI 1640 medium and incubated at 37°C in 5% CO_2_. Cells were lysed for further analyses 24 h later.

### Reporter assay

pRL-TK, pGL3-TopFlash and pEF-BOS-EX expressing candidate miRNAs were co-transfected into HEK293T cells using Lipofectamine 2000 (Invitrogen) in 24-well plate. LiCl or Wnt3a was added 6 hours after transfection for an 18-hour incubation. The luciferase activity was measured using the Dual Luciferase Reporter Assay System (Promega). Relative luciferase light units (RLUs, firefly/Renilla luciferase ratios) were reported from three independent experiments.

### Western blotting

Cells were lysed on ice in 150 μl RIPA Buffer (Beyotime biotechnology, China) supplemented with 1 mM PMSF. Protein concentration was determined using BCA Protein Assay Kit (Thermo Fisher Scientific, Rockford, USA). Equal amount of cell lysates were loaded onto SDS polyacrylamide gels. The samples were probed with anti-β-catenin (1:1000 dilution, clone 14, BD Biosciences) and anti-β-actin as a loading control. Blots were detected using SuperSignal® West Pico Chemiluminescent Substrate Kit (Product #34087, Thermo Scientific), and all signals were analyzed with ImageJ software.

### Generation of stable cell lines

MO retroviral plasmids encoding miR-142 and EGFP (miR-142) or EGFP alone (MO) were transfected into the PlatE packaging cell line, retrovirus-containing supernatant was collected 48 h later. NIH3T3 cells were infected by replacing the culture medium with the virus supernatant supplemented with 8 μg/ml Polybrene, followed by centrifugation at 1,800 rpm for 45 min. The infected cells were further cultured in medium at 37°C for 24 h, then they were selected with 1 μg/ml puromycin for 72 h. The cells were then split at a density of one cell in three wells into 96-well plates, the formed colonies were expanded and saved for further analysis.

### Cell cycle analysis

MO and miR-142 stable NIH3T3 cell lines were seeded at a density of 1×10^6^/well in a six-well dish, cultured for 24 h, then treated with or without 25 mM LiCl for 12 h. Cells were harvested and washed twice in cold PBS, fixed with 70% cold ethanol overnight at 4°C, washed with PBS again, then the cells were incubated in 50 μg/ml propidium iodide (PI)-staining solution (PBS containing 100 μg/ml of RNase A and 0.2% Triton X-100) at 4°C in dark for 30 min. The cell cycle distributions were measured using a FACSCalibur cytometer and analyzed using FACS analysis software (FlowJo).

### Statistical analyses

Data were analyzed using GraphPad Prism 5.0 Software and presented as means ± SEM. Statistical analyses were performed with the Student’s t test (p value). P < 0.05 (*), p < 0.01 (**) and p < 0.001 (***) were considered significant.

## Results

### miR-142 negatively regulates Wnt/β-catenin signaling

**Fig 1:**
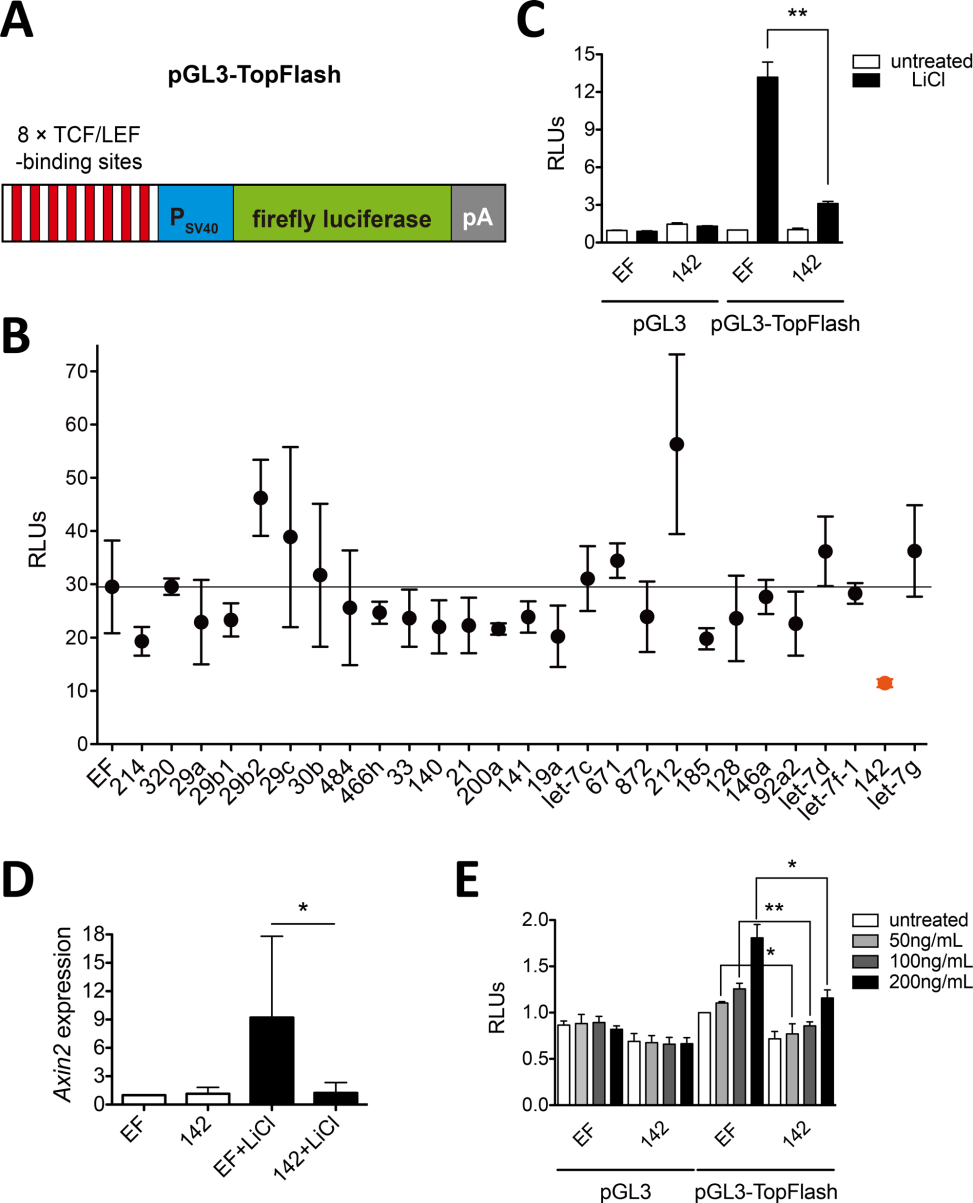
Among candidate miRNAs, miR-142 negatively regulates Wnt/β-catenin signaling. (A) Schematic representation of the pGL3-TopFlash reporter. Eight tandem repeats of TCF/LEF response elements were introduced upstream of the SV40 promoter and firefly luciferase gene (Luc) in pGL3 promoter vector. (B) Screening for inhibitors of Wnt/β-catenin signaling. Plasmids pGL3-TopFlash, pRL-TK and miRNA-expressing vectors were cotransfected into HEK293T cells. Cells were treated with 25 mM LiCl 6 h posttransfection, and lysed 24 h posttransfection for dual-luciferase analysis (RLUs, Fluc/Rluc). (C, E) miR-142 inhibits the activated Wnt/β-catenin signaling. Wnt/β-catenin signaling was activated by 25 mM LiCl (C) or increasing doses of Wnt3a (E). TopFlash-mediated firefly luciferase activities (B, C, E) were normalized to the activity of Renilla luciferase (pRL-TK); error bars mark the SEM (n = 3; **P < 0.01, *P < 0.05, t test). (D) miR-142 represses expression of *Axin2*. miR-142 expressing vector or empty vector (EF) was transfected into HEK293T cells with or without 25 mM LiCl treatment. *Axin2* mRNA expression was analyzed by quantitative RT-PCR, the data shown were normalized by *Actb* expression; error bars mark the SEM (n = 2; *P < 0.05, t test).

To identify the miRNAs that potentially modulate Wnt/β-catenin signaling, twenty-seven miRNAs, which highly expressed in T cells [4], were selected for a luciferase reporter based screening. These miRNAs expressed through cloning their pre-miRNAs plus ~ 700 bp flanking genomic sequences downstream of the EF-1a promoter, could efficiently repress the luciferase activities of individual microRNA sensor (S1 Fig). A pGL3-TopFlash reporter containing eight tandem repeats of TCF response elements was generated and used to monitor the Wnt/β-catenin signaling (Fig 1A). We determined the effects of candidate miRNAs on pGL3-TopFlash reporter after co-transfected to HEK293T cells in the presence of LiCl, which could inhibit GSK3β activity and thereby stabilize β-catenin and activate the canonical Wnt pathway [22]. As expected, LiCl treatment elevated the activity of pGL3-TopFlash reporter dramatically (Fig 1C). Half of the candidate miRNAs slightly decreased the readout of pGL3-TopFlash reporter by approximately 20–30% under LiCl treatment (Fig 1B), whereas miR-29b, miR-29c and miR-212 increased the luciferase expression, most strikingly, miR-142 reduced the luciferase activity of pGL3-TopFlash reporter by approximately 70% (Fig 1B and 1C). Overexpression of mir-142 also repressed the induction of *Axin2* (Fig 1D), which is a well documented downstream target of Wnt/β-catenin signaling [2]. Thus, miR-142 was selected for further analysis.

Next, we used Wnt3a, a naturally occurring ligand of Wnt/β-catenin signaling, to activate the pGL3-TopFlash reporter. The luciferase activities of pGL3-TopFlash reporter were increased upon Wnt3a stimulation in a dose-dependent manner, whereas such effects were blocked by miR-142 (Fig 1E). Taken together, these results indicated that miR-142 was a potential suppressor of Wnt/β-catenin signaling, which is inconsistent with a recently study reported that miR-142-3p activated the canonical WNT signaling pathway in human breast cancer stem cells through suppression of APC [16].

### Stem-loop structure of pre-miR-142 is central to the function of miR-142 gene

**Fig 2:**
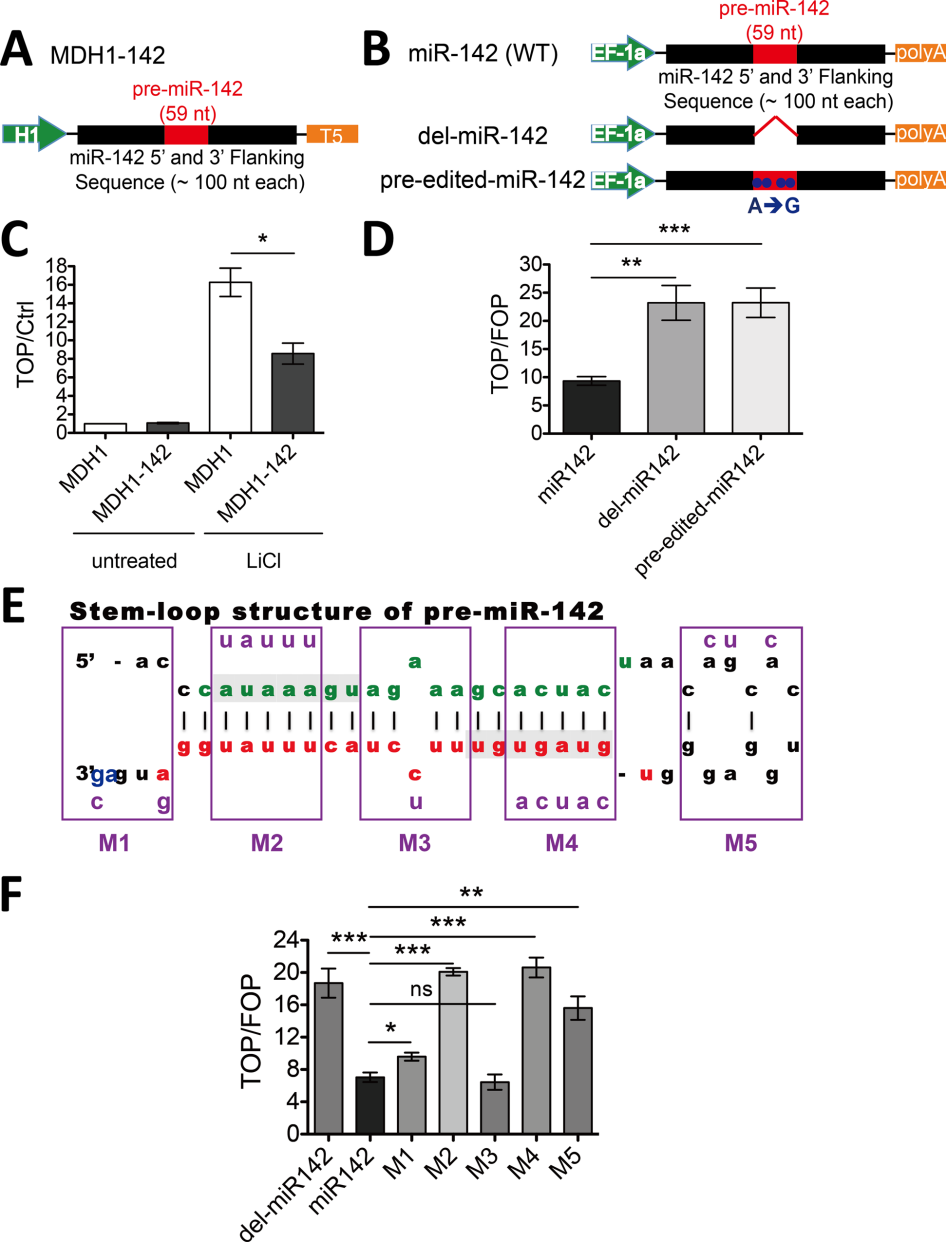
Pre-miR-142 is essential for suppressing Wnt/β-catenin signaling. A and B, schematic of the MDH1-142 (A) and EF-miR-142, EF-del-miR-142, EF-pre-edited-miR-142 (B) constructs. Pre-miR-142 (59 nt) plus flanking sequences were introduced downstream of the H1 or EF-1a promoter, followed by T5 or polyA termination signal. Whole 59 nucleotides of pre-miR-142 were deleted in the del-miR-142 construction. Four Adenosine residues within pre-miR-142 were substituted by guanosine residues (A➔G) constructed the pre-edited-miR-142 plasmid. (C) HEK293T cells were transfected with MDH1-142 expression vector or control MDH1 vector along with the pGL3-TopFlash or pGL3 empty vector and the pRL-TK vector as a normalization control. Cells were treated with 25 mM LiCl and lysed 24 h later for dual-luciferase analysis. Normalized TopFlash values were further divided by normalized pGL3 control values; error bar mark the SEM (n = 3; *P < 0.05, t test). (D) HEK293T cells were transfected with EF-miR142 expression vector or miR-142-mutant vectors along with the pGL3-TopFlash or pGL3-FopFlash vector and the pRL-TK vector as a normalization control. Normalized TopFlash values were further divided by normalized FopFlash values; error bars mark the SEM (n = 3; ***P < 0.001, **P < 0.01, t test). (E) Schematic diagram showing the structure of pre-miR-142. The part highlighted in green indicates miR-142-5p, and in red shows miR-142-3p. Bases with light-gray background represent the seed sequences of these two distinct miRNAs. The frames above M1 to M5 mark the positions of five structure-changing mutants within pre-miR-142, purple bases within the frames indicate the mutant sequences. (F) TopFlash-mediated reporter assay was performed as described in (D) with M1 ~ M5 mutants; error bars mark the SEM (n = 3; ***P < 0.001, **P < 0.01, *P < 0.05, ns P > 0.05, t test).

Due to the inconsistence with the observation by Taichi Isobe et al. [16], we performed the pGL3-TopFlash reporter assay by using a published construct MDH1-142 [21], in which ~ 250 bp genomic sequence encompassing pre-miR-142 was driven by H1, a RNA polymerase III (PolIII) promoter (Fig 2A). Similar decreased Wnt/β-catenin signaling was observed when pGL3-TopFlash reporter was co-transfected with MDH1-142 (Fig 2C). Notably, deletion of the pre-miR-142 sequence from the miR-142 expression construct did lead to release of the suppression of pGL3-TopFlash reporter activity as shown in Fig 2B and 2D. A previous study by Weidong Yong et al. had shown that A to I RNA editing of miR-142 by ADAR inhibited its processing by Drosha, resulting in a substantial reduction in mature miR-142-5p and miR-142-3p levels [23]. Thus, we substituted 4 sites of adenosine (A) with guanosine (G) on our miR-142 expressing vector (pre-edited-miR-142, S1 Table) to synthesize artificial edited pri-miR-142 RNA in transfected 293T cells. The pGL3-TopFlash signal of this pre-edited-miR-142 recovered similarly to that of del-miR-142 demonstrated that mature miR-142-5p and/or miR-142-3p were indeed responsible for repressing Wnt/β-catenin signaling. This notion was further supported by more detailed mutagenesis study. We generated a series of mutations, M1 ~ M5 (Fig 2E and S1 Table), which potentially changed the RNA secondary structure of pre-miR-142. M1 and M3, which unlikely affected the stem-loop structure of pre-miR-142, still kept the inhibitory effects of miR-142, whereas other mutations clearly lost their inhibitory effects of miR-142 (Fig 2F), thus the stem-loop structure of pre-miR-142 was critical for its function as a suppressor of the Wnt/β-catenin signaling.

### miR-142-3p but not 5p represses Wnt/β-catenin signaling pathway

**Fig 3:**
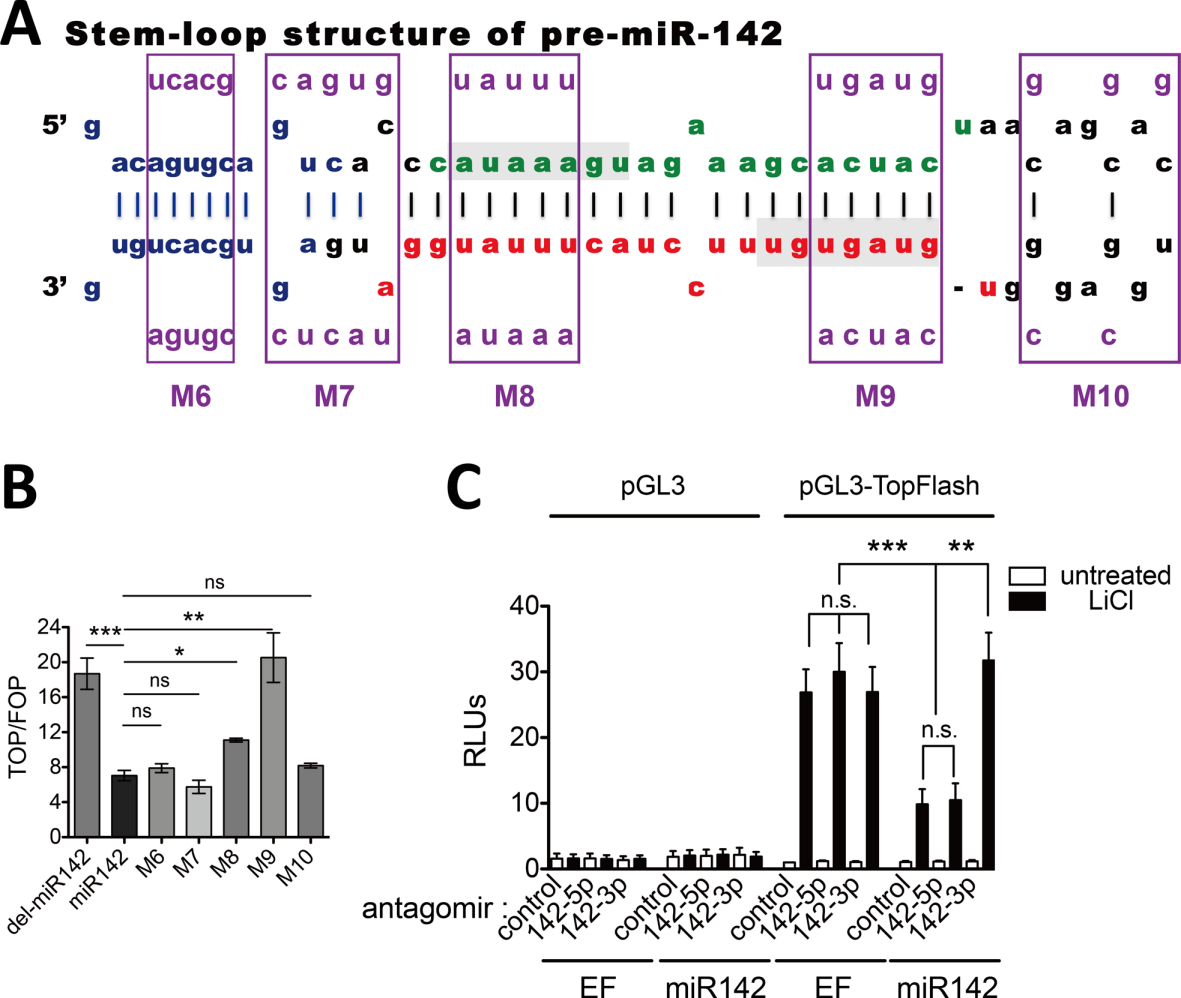
miR-142-3p suppresses Wnt/β-catenin signaling. (A) Schematic diagram showing the structure of pre-miR-142. The part highlighted in blue indicates sequences surrounding the pre-miR-142. The frames above M6 to M10 mark the positions of five structure-unchanging mutants within or near pre-miR-142, purple bases within the frames indicate the mutant sequences. (B) TopFlash-mediated reporter assay was performed as described in Fig 2D with M6 ~ M10 mutants; error bars mark the SEM (n = 3; ***P < 0.001, **P < 0.01, *P < 0.05, ns P > 0.05, t test). (C) Antagomir-142-3p abolished the effects of miR142. TopFlash mediated reporter assay was performed as in Fig 1C. Antagomir-142-5p, antagomir-142-3p or control antagomir was added, respectively. TopFlash-mediated firefly luciferase activities were normalized to that of Renilla luciferase; error bars mark the SEM (n = 3; ***P < 0.001, **P < 0.01, t test).

The pre-miR-142 is processed to two mature microRNAs, miR-142-5p and/or 3p (Fig 3A), depending on which arm is loaded into the RNA-induced silencing complex (RISC). It has been reported that both miR-142-5p and miR-142-3p are expressed in hematopoietic cell lineages [4]. To investigate which mature miRNAs of miR-142 gene is responsible for the repression of Wnt/β-catenin signaling, we constructed another series of mutants of miR-142 expressing vector, M6 ~ M10 (Fig 3A and S1 Table). These mutations were located within the seed sequence of miR-142-5p (M8) or miR-142-3p (M9), the loop region (M10), or flanking sequences (M6 and M7). Seed sequences and their complementary strains were mutated simultaneously according to the principle of complementary base pairing to keep the RNA secondary structure of pre-miR-142 intact. M6, M7 and M10 kept the inhibitory effects of miR-142, indicating the flanking parts and the loop region were not essential for the function of miR-142. Interestingly, the M9 mutant significantly released the inhibitory effect of miR-142 expressing vector whereas M8 did not, which highlighted that miR-142-3p was responsible for repressing Wnt/β-catenin signaling (Fig 3B).

We further confirmed the functional difference between miR-142-5p and 3p by using specific antagomirs to antagonize each miRNA (S2 Fig). As shown in Fig 3C, inhibition of miR-142-3p instead of miR-142-5p, resulted in recovery of the luciferase activity. Together, these results indicated that the inhibitory effect of miR-142 on Wnt/β-catenin signaling was specifically mediated by miR-142-3p but not 5p.

### miR-142-3p directly binds to *Ctnnb1* 3’ UTR and affects β-catenin translation

**Fig 4:**
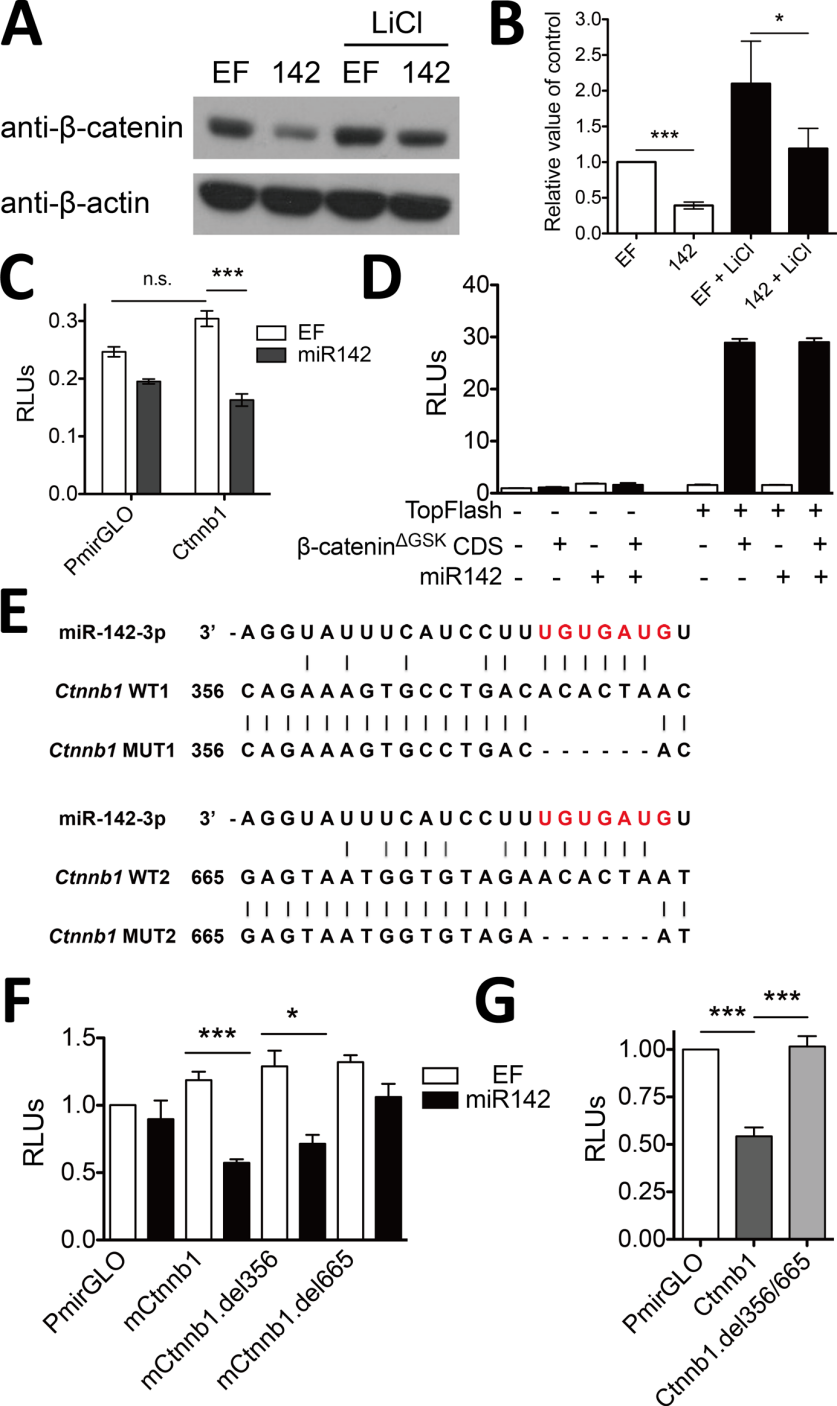
miR-142 directly targets *Ctnnb1* mRNA and decreases β-catenin protein level. (A) Ectopic expression of miR-142 decreased β-catenin protein level. miR-142 expressing vector or empty vector (EF) was transfected into HEK293T cells with or without 25 mM LiCl treatment. (B) Quantitative analysis of the densitometric values of β-catenin signal relative to β-actin; error bars mark the SEM (n = 5; ***P < 0.001, *P < 0.05, t test). (C) miR-142 directly targeted 3’ UTR of β-catenin. Mouse *Ctnnb1* 3’ UTR was introduced downstream of the luciferase gene in the PmirGLO vector. This vector and miR-142 expressing vector were co-transfected into HEK293T cells. PmirGLO is used as a control. (D) miR-142 did not target CDS of β-catenin. Construct expressing β-catenin^ΔGSK^ mutant was co-transfected with pGL3-TopFlash, miR-142 expressing vector, and pRL-TK into HEK293T cells. (E) Schematic representation of two putative miR-142-3p binding sites within mouse *Ctnnb1* 3’ UTR. Bases highlighted in red indicate the seed sequence of miR-142-3p, and lines in black indicate base pairings. (F) nt665-671 of mouse *Ctnnb1* 3’ UTR was essential for the regulation by miR-142-3p. PmirGLO or its variant expressing wild type *Ctnnb1* 3’ UTR or mutated *Ctnnb1* 3’ UTR (nt356-361- or nt665-671-deletion) was cotransfected with miR-142 expressing vector into HEK293T cells, respectively. (G) Endogenous miR142 targeted to synthetic *Ctnnb1* 3’ UTR in Jurkat T cells. PmirGLO or its variant expressing wild type *Ctnnb1* 3’ UTR or mutated *Ctnnb1* 3’ UTR (nt356-361- and nt665-671-deletion) was electransfected to Jurkat T cells, respectively. Luciferase activities (C, D, F, G) were assessed 24 h after transfection and normalized to the activity of Renilla luciferase; error bars mark the SEM (n = 3; ***P < 0.001, *P < 0.05, t test).

To investigate whether miR-142 directly regulates β-catenin, we overexpressed miR-142 in HEK293T cells and stimulated these cells with 25 mM LiCl. The β-catenin protein levels were determined by western blotting. As expected, LiCl induced the accumulation of β-catenin though inhibition of GSK3β. Importantly, less β-catenin protein were present in miR-142-expressing cells than that in control cells (Fig 4A and 4B) while mRNA of *Ctnnb1* maintained a constant level (S3 Fig) both present and absent of LiCl treatment, suggesting that miR-142 posttranscriptionally decreased endogenous β-catenin protein level in both quiescent and active status of Wnt/β-catenin signaling pathway. To further confirm that the β-catenin transcript was directly targeted by miR-142, we cloned the 3’ UTR sequence of *Ctnnb1* into the PmirGLO reporter downstream of the firefly luciferase gene to generate PmirGLO-Ctnnb1 reporter. Reporter assay showed that cells overexpressing miR-142 had 50% lower readout of PmirGLO-Ctnnb1 reporter than that of control (Fig 4C and S4 Fig), demonstrating that miR-142 directly targeted the 3’ UTR of *Ctnnb1*. Moreover, pGL3-TopFlash luciferase assay of cells expressing miR-142 and β-catenin^ΔGSK^ mutant showed that miR-142 was unable to decrease the transcriptional activity of β-catenin when its 3’ UTR was missing (Fig 4D).

Next, alignment analysis showed that the *Ctnnb1* 3’ UTR sequence bore two putative miR-142-3p binding sites, nt356-361 and nt665-671 (Fig 4E). To evaluate the direct interaction with miR-142-3p, we deleted each putative miR-142-3p binding site (*Ctnnb1*.del356 or *Ctnnb1*.del665) from the PmirGLO-Ctnnb1 vector. As a result, deletion of nt665-671 eliminated miR-142-3p-mediated repression, which is similar to the effect of the M9 mutant of pre-miR-142 (Fig 3B), while deletion of nt356-361 did not affect the outcome of miR-142 overexpression (Fig 4F). These results indicated that miR-142-3p acted as a repressor of β-catenin expression by directly binding to nt665-671 of *Ctnnb1* 3’ UTR.

We also examined the effects of endogenous miR-142 on PmirGLO-Ctnnb1 reporter in Jurkat cells, a human T lymphocyte cell line. Similarly, endogenous miR-142 in Jurkat cells (S5 Fig) inhibited 50% of the luciferase activity of the wild-type *Ctnnb1* 3’ UTR reporter, but neither the control nor the mutant del356/665 *Ctnnb1* 3’ UTR reporter (Fig 4G). Together, these data supported the notion that miR-142-3p could directly interact with *Ctnnb1* 3’ UTR to inhibit the canonical Wnt/β-catenin signaling.

### miR-142 inhibits cell proliferation through regulating Wnt/β-catenin signaling

**Fig 5:**
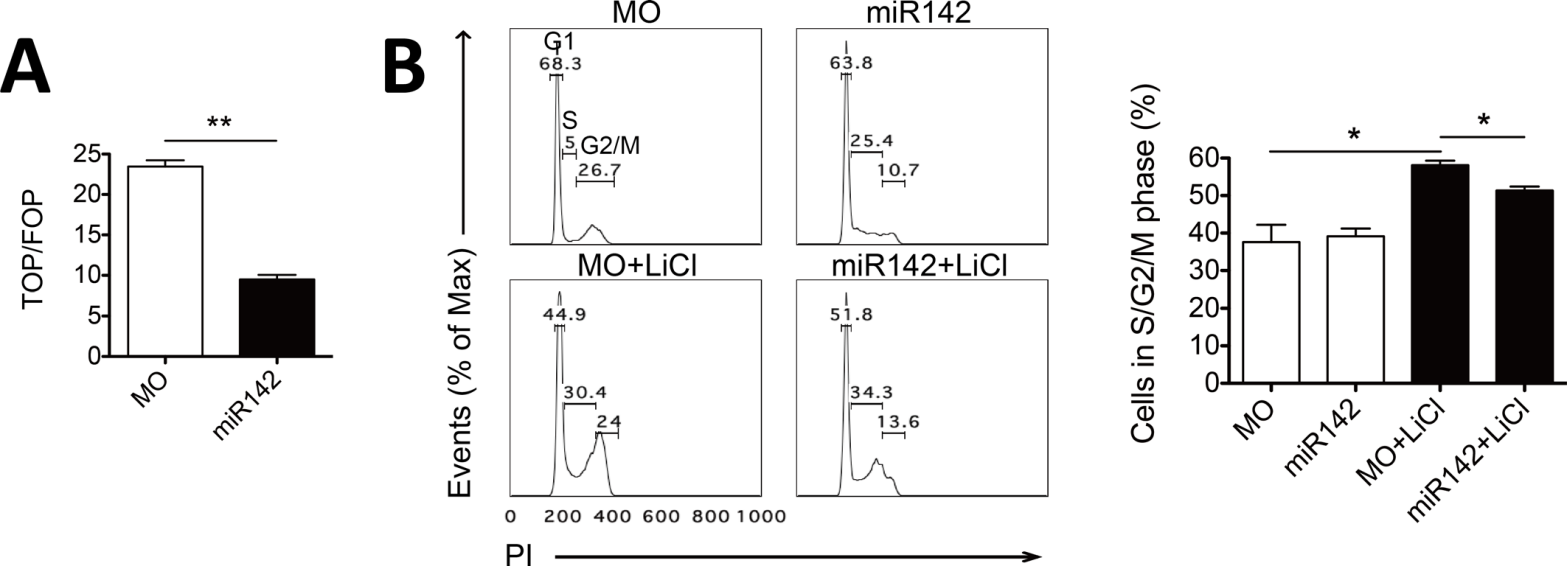
miR-142 represses cell proliferation. (A) miR-142 and control (MO) stable NIH3T3 cell lines were transfected with the pGL3-TopFlash or pGL3-FopFlash vector and the pRL-TK vector as a normalization control. Cells were treated with 25 mM LiCl and lysed 24 h later for dual-luciferase analysis. Normalized TopFlash values were further divided by normalized FopFlash values; error bars mark the SEM (n = 2; **P < 0.01, t test). (B) Representative FACS histograms of propidium iodide (PI) staining of miR-142 and control (MO) stable NIH3T3 cell lines treated with or without LiCl (left) and the quantified percentages of cells in the S and G2/M phases respectively (right); error bars mark the SEM (n = 2; *P < 0.05, t test).

Wnt/β-catenin signaling is involved in cell proliferation and survival [2], to investigate whether miR-142 has a role in cell cycle regulation, we constructed miR-142-expressing-stable NIH3T3 cell lines (miR-142) and control cell lines (MO) and measured the cell cycle distributions. Consistently, resulting miR-142 stable clones also displayed repressed Wnt/β-catenin signaling under LiCl treatment compared to MO clones (Fig 5A). Propidium iodide (PI) staining showed that at steady state, the cell cycle distributions of miR-142 and MO clones had little differences. LiCl induced cell cycle progression as the S and G2/M phases of MO cells increased by 20%. However, expression of miR-142 led to rather less cells in the S and G2/M phases compared to MO clones under LiCl treatment (Fig 5B), suggesting miR-142 is able to inhibit cell proliferation by repressing active Wnt/β-catenin signaling.

## Discussion

Both loss- and gain-of-function mutations of β-catenin are associated with numerous cancers or developmental perturbations [12, 13, 24–31], implying a critical window for β-catenin expression level. It is well known that the abundance of β-catenin is tightly controlled by destruction complex in the cytoplasm. Mutations in any component of this complex will lead to excessive accumulation of β-catenin and constitutive activation of the Wnt pathway. In addition, several miRNAs have been demonstrated to target different components of Wnt signaling pathway [14, 15, 32–38]. In this study, we reported miR-142-3p could negatively regulate Wnt/β-catenin signaling through direct posttranscriptional repression of β-catenin, which provides new insights into the regulation of Wnt/β-catenin signaling pathway.

Although it was recently suggested by Taichi Isobe et al. that miR-142-3p activates the canonical WNT signaling pathway through suppression of APC in human breast cancer stem cells [16]. Amit Shrestha et al. found increased protein level of β-catenin while *Ctnnb1* mRNA is not altered in the miR-142-null lungs, indicating Wnt signaling was increased in the miR-142 deficient mice [39]. Therefore, we speculate that miR-142-3p could play dual roles in fine-tuning Wnt signaling in a context dependent manner, when miR-142 has low expression, it might only target APC, whereas high concentrated miR-142 represses expression of both APC and β-catenin. Thus, the eventual outcome of miR-142-3p on Wnt signaling may diverse among tissues and cells depending on which mRNA are trapped more by miR-142-3p under specific cell circumstances. Further investigation is needed to illustrate this possibility on each specific case.

MiR-142 is among the first miRNAs found to exclusively express in hematopoietic cells [21]. Its deficiency leads to splenomegaly and a slight infiltration of lymphoid organs by myeloid cells, mainly neutrophils [7]. Moreover, miR-142-3p is important for the specification and differentiation of HSC lineage during embryogenesis [40, 41]. Knockdown of this miRNA results in reduced HSCs as well as T-cell defect in zebrafish and mouse [40, 42]. Similarly, constitutively-active-β-catenin-expressing lymphoid progenitors fail to produce T cells [43]. In addition, both ablation of miRNA [8] and activation of the Wnt signaling [44] result in dysfunction of regulatory T (T reg) cells. The similar phenotypes caused by miR-142 deficiency and gain- of function of Wnt/β-catenin signaling are consistent with our finding demonstrating the inhibitory effect of miR-142-3p on Wnt/β-catenin signaling. Taken together, Our results demonstrate that miR-142-3p is a new suppressor of β-catenin and could be a candidate target for future tumor therapy [45].

## Supporting Information

S1 Fig MicroRNAs specifically target cognate sensors in this study. PmirGLO vectors with none (PmirGLO), reverse complement sequences of miR-142-3p (T142-3p), miR-142-5p (T142-5p), miR-200a-3p (T200a-3p) and miR-142, miR200a expressing vectors or empty vector were cotransfected to HEK293T cells and luciferase activities were assessed 24 h after transfection. Firefly activities were normalized to the activity of Renilla luciferase; error bars mark the SEM (n = 3; *P < 0.05, t test).
(PDF)

S2 Fig Specific inhibition of miRNA by antagomir. (A-B) HEK293T cells were transfected with PmirGLO-T142-3p (A) or PmirGLO-T142-5p (B), and miR-142 expressing vector or empty vector, plus antagomir control, antagomir-142-5p and antagomir-142-3p. Luciferase activities were assessed 24 h after transfection. Firefly activities were normalized to the activity of Renilla luciferase; data are representative of two experiments.
(PDF)

S3 Fig *Ctnnb1* mRNA maintains a constant level in miR-142 overexpressing HEK293T cells. Shown is qPCR analysis of *Ctnnb1* in miR-142 expressing HEK293T cells or control cells (EF) with or without 25 mM LiCl treatment.
(PDF)

S4 Fig miR-142-3p directly targets β-catenin 3’UTR. HEK293T cells were transfected with PmirGLO-Ctnnb1 and miR-142 expressing vector or empty vector, plus antagomir control, antagomir-142-5p and antagomir-142-3p. Luciferase activities were assessed 24 h after transfection. Firefly activities were normalized to the activity of Renilla luciferase; error bars mark the SEM (n = 2; *P < 0.05, t test).
(PDF)

S5 Fig Both miR-142-3p and miR-142-5p are expressed in Jurkat cells. Jurkat cells were electransfected with PmirGLO vectors with none (PmirGLO), reverse complement sequences of miR-142-5p (T142-5p), miR-142-3p (T142-3p), miR-200a-3p (T200a-3p) and assessed for luciferase activities 24 h after transfection. Firefly activities were normalized to the activity of Renilla luciferase; error bars mark the SEM (n = 3; *P < 0.05, **P < 0.01, t test).
(PDF)

S1 Table Structures and sequences of WT and mutants of pre-miR-142. (PDF)
