# Supplementary figures and images for: MicroRNA-142-3p Negatively Regulates Canonical Wnt Signaling Pathway
Source: PLoS One. 2016 Jun 27;11(6):e0158432. doi: 10.1371/journal.pone.0158432 (PMC4922628; doi:10.1371/journal.pone.0158432)

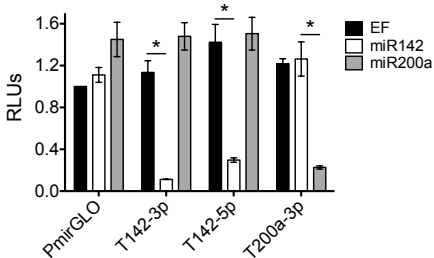

Supplement: S1 Fig — PmirGLO vectors with none (PmirGLO), reverse complement sequences of miR-142-3p (T142-3p), miR-142-5p (T142-5p), miR-200a-3p (T200a-3p) and miR-142, miR200a expressing vectors or empty vector were cotransfected to HEK293T cells and luciferase activities were assessed 24 h after transfection. Firefly activities were normalized to the activity of Renilla luciferase; error bars mark the SEM (n = 3; *P < 0.05, t test). (PDF) [file pone.0158432.s001.pdf]

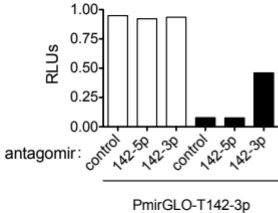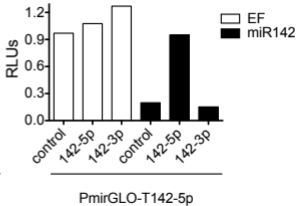

Supplement: S2 Fig — (A-B) HEK293T cells were transfected with PmirGLO-T142-3p (A) or PmirGLO-T142-5p (B), and miR-142 expressing vector or empty vector, plus antagomir control, antagomir-142-5p and antagomir-142-3p. Luciferase activities were assessed 24 h after transfection. Firefly activities were normalized to the activity of Renilla luciferase; data are representative of two experiments. (PDF) [file pone.0158432.s002.pdf]

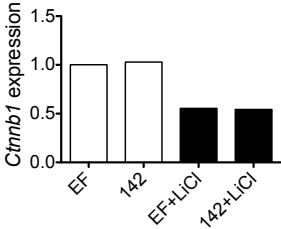

Supplement: S3 Fig — Shown is qPCR analysis of Ctnnb1 in miR-142 expressing HEK293T cells or control cells (EF) with or without 25 mM LiCl treatment. (PDF) [file pone.0158432.s003.pdf]

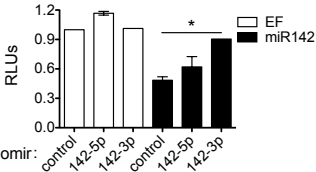

PmirGLO-Ctnnb1

Supplement: S4 Fig — HEK293T cells were transfected with PmirGLO-Ctnnb1 and miR-142 expressing vector or empty vector, plus antagomir control, antagomir-142-5p and antagomir-142-3p. Luciferase activities were assessed 24 h after transfection. Firefly activities were normalized to the activity of Renilla luciferase; error bars mark the SEM (n = 2; *P < 0.05, t test). (PDF) [file pone.0158432.s004.pdf]

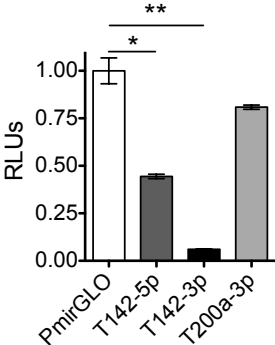

Supplement: S5 Fig — Jurkat cells were electransfected with PmirGLO vectors with none (PmirGLO), reverse complement sequences of miR-142-5p (T142-5p), miR-142-3p (T142-3p), miR-200a-3p (T200a-3p) and assessed for luciferase activities 24 h after transfection. Firefly activities were normalized to the activity of Renilla luciferase; error bars mark the SEM (n = 3; *P < 0.05, **P < 0.01, t test). (PDF) [file pone.0158432.s005.pdf]
