# Supplementary material for: MicroRNA-142-3p Negatively Regulates Canonical Wnt Signaling Pathway
Source: PLoS One. 2016 Jun 27;11(6):e0158432. doi: 10.1371/journal.pone.0158432 (PMC4922628; doi:10.1371/journal.pone.0158432)
Supplement: S1 Table — (PDF) [file pone.0158432.s006.pdf]

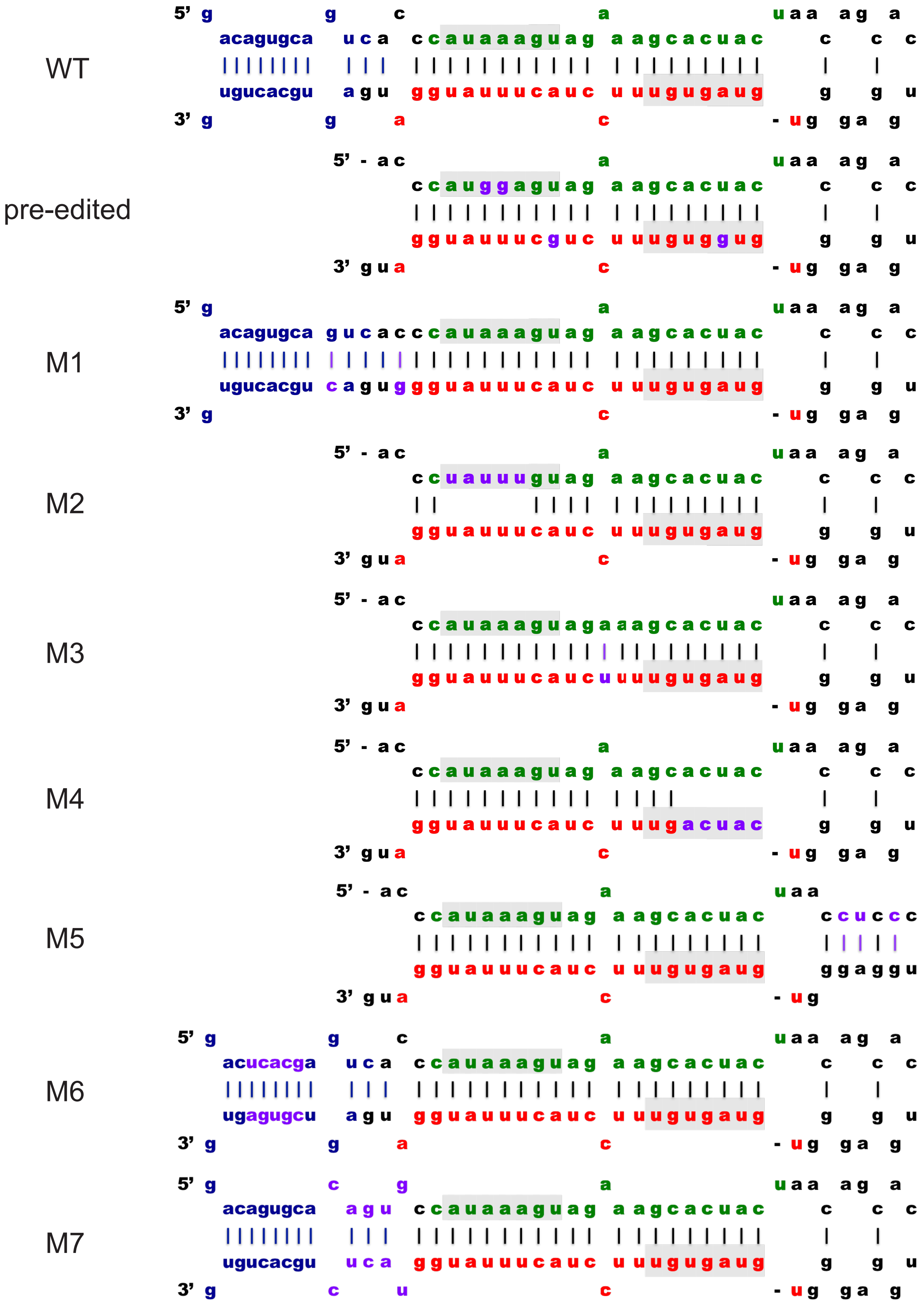
WT
pre-edited
M1
M2
M3
M4
M5
M6
M7

M8

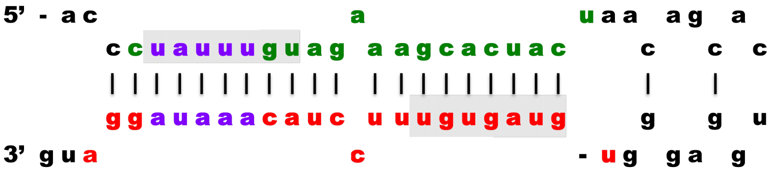
5' - ac a uaa ag a
ccuauuuguag aagcacuac c c c
gguaaacauc uugugaug g g u
3' gua c -ug ga g

M9

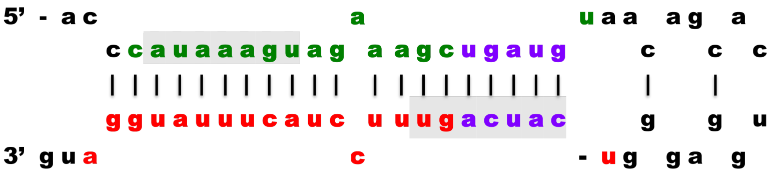
5' - ac a uaa ag a
ccauaaaguag aagcugaug c c c
gguauuucauc uugacuac g g u
3' gua c -ug ga g

M10

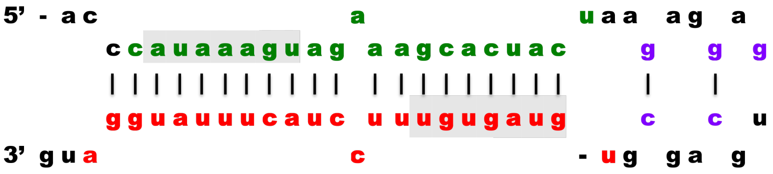
5' - ac a uaa ag a
ccauaaaguag aagcacuac g g g
gguauuucauc uugugaug c c u
3' gua c -ug ga g
